# Nanozyme-Based Strategies in Cancer Immunotherapy: Overcoming Resistance to Enhance Therapeutic Efficacy

**DOI:** 10.14336/AD.2025.0011

**Published:** 2026-02-14

**Authors:** Guangjian Hou, Yukun Xu, Chunhua Wang, Can Lu, Abhimanyu Thakur, Kui Zhang, Wei Li, Zhijie Xu

**Affiliations:** ^1^The First Clinical Medical College of Shandong University of Traditional Chinese Medicine, Jinan, Shandong, China.; ^2^Department of Geriatric Medicine, Affiliated Hospital of Shandong University of Traditional Chinese Medicine, Jinan, Shandong, China.; ^3^Department of Oral Bioscience, Tokushima University Graduate School of Biomedical Sciences, Tokushima, Japan.; ^4^Department of Pathology, Xiangya Hospital, Central South University, Changsha, Hunan, China.; ^5^National Clinical Research Center for Geriatric Disorders, Xiangya Hospital, Central South University, Changsha, Hunan, China.; ^6^Pritzker School of Molecular Engineering, Ben May Department for Cancer Research, University of Chicago, Chicago, IL 60637, USA.; ^7^Nephropathy Department, Affiliated Hospital of Shandong University of Traditional Chinese Medicine, Jinan, Shandong, China

**Keywords:** nanomaterials, nanozyme, immunotherapy, immune response, cancer

## Abstract

Nanozymes, which are nanomaterials that replicate the catalytic activities of natural enzymes in biological systems, have recently demonstrated considerable potential in improving cancer immunotherapy by altering the tumor microenvironment. Nanozyme-driven immune responses represent an innovative therapeutic modality with high effectiveness and minimal side effects. These nanozymes activate the immune system to specifically recognize and destroy cancer cells. Combined with immunotherapeutic agents, nanozymes can amplify the anti-cancer effectiveness by integrating immune remodeling with immunogenic cell death (ICD). This review offers a thorough discussion about various nanozymes involved in anti-cancer immunity, including those mimicking catalase (CAT), superoxide dismutase (SOD), peroxidase (POD), and oxidase (OXD). It also discusses the challenges and future directions for translating nanozyme platforms into clinical applications, enhancing the susceptibility of cancer cells to immunotherapy. Nanozyme-based strategies have substantial potential in oncology, offering new and effective therapeutic options for cancer management.

## 1. Introduction

Malignant tumors continue to present an insurmountable challenge and pose a serious threat to human health[1]. Surgery remains the primary treatment modality for many types of cancer. The clinical application of chemotherapy, radiation therapy, and neoadjuvant treatments has improved patient survival rates. Despite these advances, local recurrence and therapeutic resistance are the primary driver factors of cancer-related mortality [2-4]. Consequently, developing new therapeutic agents to combat cancer remains a high priority and consistently occupies a central position in research agendas.

Immunotherapy has shown potential to revolutionize cancer treatment [5, 6]. These therapeutic strategies include immune checkpoint inhibitors (ICIs), cytokines, antibodies and adoptive cell therapies (ACTs), all of which primarily stimulate T-cell-mediated immune responses against cancer [7]. Immunotherapy is based on the complex behaviors and interactions of immune components within the tumor microenvironment (TME) [8, 9]. In spite of this, its effectiveness is frequently limited in many patients due to the resistance [10, 11] or side effects [12]. The intricacies of immune cell interactions in cancer cell recognition and destruction remain crucial areas of research [13], and the underlying mechanisms are not yet fully elucidated. Enhancing our understanding and precise control of immune cytotoxicity offers an opportunity to tailor individual responses to immuno-oncology therapeutics.

**Table 1:** Nanozymes are particularly effective in mimicking activities of natural enzymes in cancer immunity.

Enzyme-like categories	Names	Substrates	Anti-tumor immunity	Cancer models	Refs.
**CAT**	CaO_2_/DOX@SiO_2_/DOX-MnO_2_	H_2_O_2_	Sensitizing cancer immunochemotherapy	B16F10 melanoma models	[40]
**CAT**	ZrO_2_-x@Pt/AIPH	H_2_O_2_	Improving ICD and ICB sensitivity	4T1 tumor-bearing mice	[44]
**CAT/POD**	Bi_2_Te_3_-Au/Pd	H2O2,3,3,5,5-tetramethylbenzidine	Inducing ICD	4T1 tumor-bearing mice	[45]
**CAT/OXD**	HABT-C	H_2_O_2_,glucose	Promoting infiltrating of immune effector T cells	4T1 tumor-bearing mice	[47]
**CAT**	Au/CuNDs-R848	H_2_O_2_	Enhancing immunogenicity of breast cancer cells	4T1 tumor-bearing mice	[48]
**CAT**	Fe-PDAP/Ce6	H_2_O_2_	Generating the sonodynamic immunotherapy	4T1 tumor-bearing mice	[49]
**CAT/SOD**	Zr-CeO	H_2_O_2_, xanthine	Promoting tumor cell death and PD-1 inhibition sensitization	Renal subcutaneous tumors	[51]
**CAT**	RhRe	H_2_O_2_	Suppressing recurrence and inflammatory response	4T1 tumor-bearing mice	[52]
**CAT/OXD**	AuPtAg-Gox	H_2_O_2_, glucose	Reversing immunogenic “cold” tumor microenvironment	4T1 tumor-bearing mice	[54]
**CAT/OXD**	gCM@MnAu	H_2_O_2_, glucose	Activating anti-cancer immunogenicity	4T1 tumor-bearing mice	[55]
**CAT/OXD**	CuCo(O)@PCN	H_2_O_2_, glucose	improving intratumoral infiltration of proinflammatory cytokines and cytotoxic T cells	4T1 tumor-bearing mice	[56]
**POD**	Cu-DBCO/CL	Cholesterol	Triggering ferroptosis, cuproptosis and ICD	4T1 tumor-bearing mice	[57]
**CAT/POD**	BiF_3_@BiOI@Pt-PVP	H_2_O_2_	Modulating immune cell infiltration and enhancing immunotherapy	4T1 tumor-bearing mice	[58]
**POD/OXD**	CSN	H_2_O_2_,L-ascorbic acid	Driving free radical generation and ICD	4T1 tumor-bearing mice	[59]
**POD/OXD**	UCCG	3,3,5,5-tetramethylbenzidine	Reversing immunosuppressive microenvironment, and improving anti-PD-L1 sensitivity	4T1 tumor-bearing mice	[60]
**POD**	CpG/Cu-LDHs	3,3,5,5-tetramethylbenzidine	synergizing CDT with ICD	GL261 tumor-bearing mice	[61]
**POD/OXD**	Cu-NS@UK@POx	H_2_O_2_	Inducing ROS storms-dependent ICD	4T1 tumor-bearing mice	[62]
**POD**	Fe-TCPP-MOF	H_2_O_2_	Prompting the liberation of TAAs and inducing ICD	B16F10 melanoma models	[67]
**POD**	PP3244@Fe-ZT	H_2_O_2_	Triggering anti-cancer immune activation	4T1 tumor-bearing mice	[68]
**POD/CAT**	BMCL	H_2_O_2_,lactic acid	Activating anti-tumor immunity	CT26 colon cancer-bearing mice	[69]
**OXD**	Syr/LOD@HFN	H_2_O_2_,3,3,5,5-tetramethylbenzidine	Facilitating oxidative stress and remodeling immune microenvironment	B16F10 melanoma models	[70]
**OXD/CAT/POD**	HCS-FeCu	3,3,5,5-tetramethylbenzidine	Sensitizing breast cancer to immunotherapy	4T1-Luc breast tumor-bearing mice	[71]
**OXD**	Fe_3_O_4_@ZIF-8/GOx@MnO_2_	Glucose,3,3,5,5-tetramethylbenzidine	Reshaping the immunosuppressive microenvironment	4T1 tumor-bearing mice	[72]
**SOD/CAT/POD/OXD**	CMZM	O_2_^-^,H_2_O_2_,5,5-dithiobis-(2-nitrobenzoic acid)	Promoting DC maturation and facilitating tumor-infiltration of cytotoxic T cells	4T1 tumor-bearing mice/	[77]
**SOD/POD**	LPZ	O_2_^-^,H_2_O_2_,3,3,5,5-tetramethylbenzidine	Eliciting intense immune activation and pyroptosis	4T1 tumor-bearing mice	[104]

**Figure 1.**
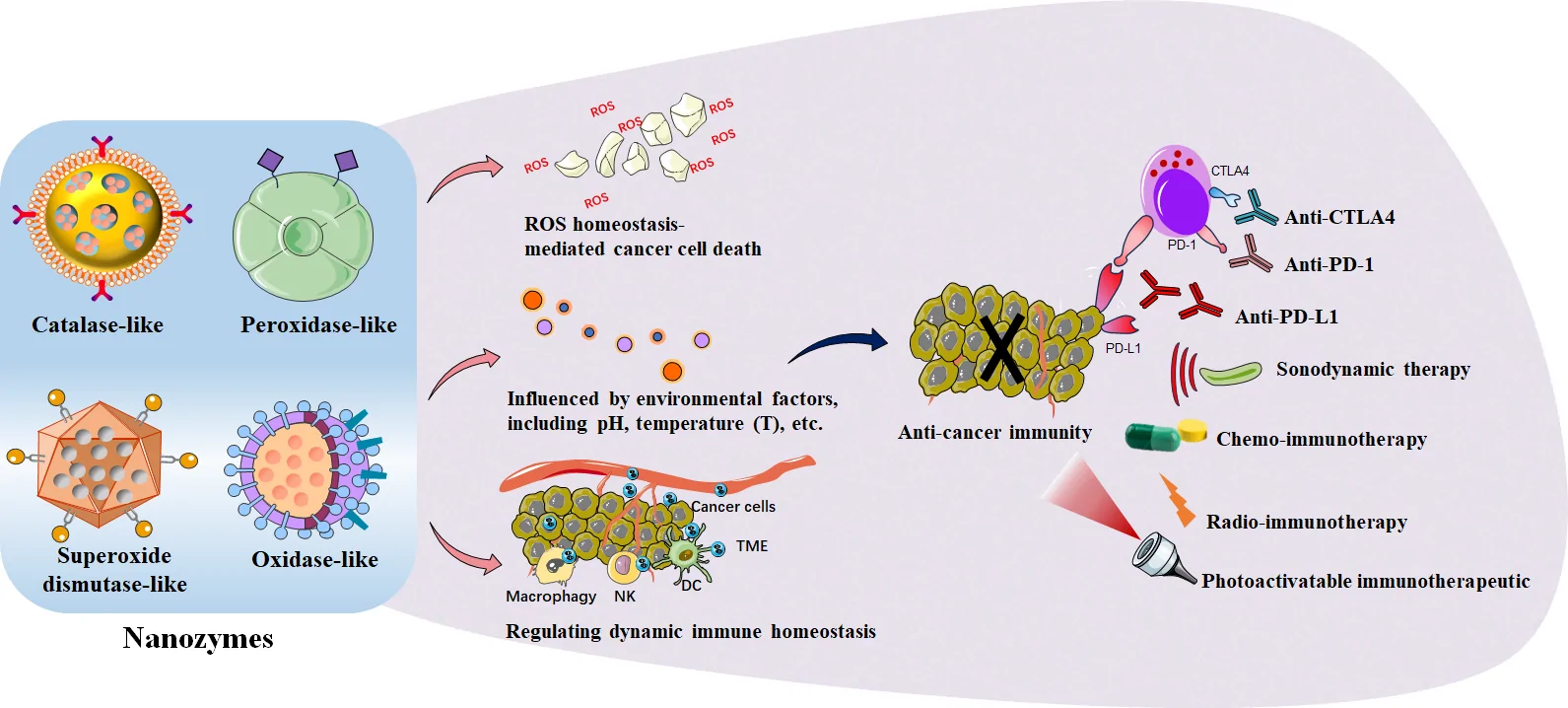
**Schematic characteristics of major nanozymes and their applicability for anti-cancer immunity**. Nanozymes are particularly effective in modulating ROS homeostasis and dynamic TME. And their activity is frequently influenced by various environmental factors, such as pH, T, and compounds in TME.

In recent years, advances in nanobiotechnology have made significant strides in diagnosing and treating cancer [14, 15]. Nano-sized materials are characterized by extensive surface areas, enhanced sensitivity, specificity, and increased reactivity for biological applications [16, 17]. As previously reported, metal-organic framework (MOF) nanoparticles, which have customizable pore sizes and high surface areas, have been successfully used to encapsulate anti-cancer agents and enhance their antagonistic actions[18-20]. Similarly, nanozymes have recently emerged as a new class of nanomaterials with enzyme-like activities [21]. Importantly, these nanozymes overcome many limitations associated with natural enzymes [22, 23]. Compared to natural enzymes, nanozymes provide numerous benefits, such as simpler synthesis processes, adjustable catalytic activity, enhanced stability, and recyclability [24, 25]. Though mimicking activities of natural enzymes, like catalase (CAT), superoxide dismutase (SOD), oxidase (OXD) and peroxidase (POD) (Table 1), nanozymes are particularly effective in modulating the oxidative stress response and maintaining reactive oxygen species (ROS) homeostasis [26, 27] (Table 2). Recent studies indicate that nanozymes are a promising avenue for tailoring individual responses to immuno-oncology therapeutics. In cancer management, they serve as efficient immunomodulatory agents because of their capacity to regulate dynamic TME and block immune escape [28, 29]. A seminal report by Zhu’s group [30] revealed that tumor-targeting nanozymes enhance the anti-cancer effects of chimeric antigen receptor T-cell immunotherapy (CAR-T) by remodeling the immune-hostile cancer micro-environment. Though integrating a semiconducting polymer core with kynureninase, the activatable polymer nanoenzyme SPNK could facilitate the singlet oxygen (^1^O_2_) generation and effector T-cell infiltration, ultimately driving immunogenic cell death (ICD) in cancer [31]. These studies demonstrate the potential of nanozyme-based models to regulate the diversity of immune environments, enhancing their correlation with the clinical responses of immunotherapy (Fig. 1).

**Table 2:** The nanozymes for generating or scavenging ROS in cancer immunotherapy.

Names	ROS-based Functions	Cancers	Refs.
**ZrO_2_-x@Pt/AIPH**	The yield of ROS indicates an attractive anti-tumor immunity	Breast cancer	[44]
**Bi_2_Te_3_-Au/Pd**	Photothermal-augmented ROS generation, and inducing ICD	Breast cancer	[45]
**HABT-C**	Facilitating ROS production, and inhibiting immunosuppressive mediators	Breast cancer	[47]
**Au/CuNDs-R848**	Mitigating ROS scavenging and enhancing the immunity	Breast cancer	[48]
**Fe-PDAP/Ce6**	Producing ROS and releasing TAAs	Breast cancer	[49]
**Zr-CeO**	Scavenging ROS and hindering immunosuppression microenvironment	Renal cancer	[51]
**RhRe**	Scavenging ROS for anti-inflammatory treatment	Breast cancer	[52]
**Cu-DBCO/CL**	Catalyzing O2 and H2O2 to ROS, and restoring antitumor activity of T cells	Breast cancer	[57]
**BiF_3_@BiOI@Pt-PVP**	Facilitating cascade reaction of ROS, and improving ROS generation	Breast cancer	[58]
**UCCG**	Boosting ROS production and reversing suppressive TME,	Breast cancer	[60]
**BMCL**	Continuous release of H_2_O_2_ and inducing anti-tumor immunity	Colon cancer	[69]
**HCS-FeCu**	Improving ROS production and immumotherapy	Breast cancer	[71]
**LPZ**	Generating ROS and intense immune activation	Breast cancer	[104]

Recent discoveries in this field offer a comprehensive perspective on the mechanisms of nanozymes in cancer immunity and suggest valuable directions for overcoming the limitations of current therapies. In this review, we give an up-to-date summarize about the impact of nanozyme modifications on anti-cancer immunity, including clinical trial results that highlight its promising clinical significance. We summarize known signal biomarkers and emerging technologies that could assess and predict the efficacy of nanozyme-based methods, potentially inspiring novel research avenues and opportunities to refine immunotherapy.

## 2. The main nanozymes with anti-cancer immune regulation activity

As a promising strategy, catalytic therapy could directly deliver the specific agents into tumor sites. Since the identification of nanozymes in 2007, significant advancements have been made in their application for cancer research and treatment [32]. Nanozymes are highly adaptable to TME, and could obviously alleviate hypoxia though facilitating the generation of toxic ROS or O_2_, thus driving the anti-cancer catalytic therapy [33]. Therefore, these nanozymes are vital for regulating TME and boosting the effectiveness of immunotherapy.

### 2.1. Catalase-like nanozymes

**Figure 2.**
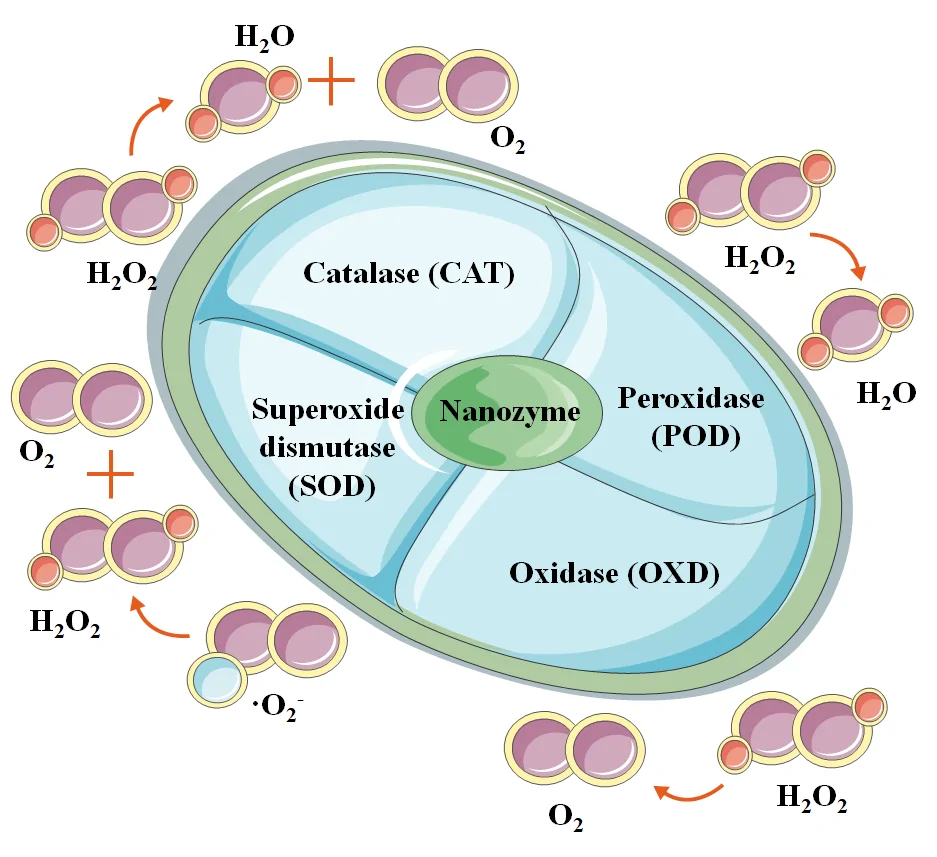
Schematic categories of major nanozyme that mediate reactive oxygen species (ROS) homeostasis and oxidative stress response for cancer immunotherapy, including CAT-, POD-, and OXD- and SOD-mimicking nanozymes.

Catalases, a family of anti-oxidant enzymes, are ubiquitous in all oxygen-exposed organisms, including plants and animals [34]. Generally, the overproduction of ROS disrupts redox homeostasis, thereby causing oxidative stress and ROS-induced damage to critical biomolecules in cells, such as nucleic acids, proteins, and membranes [35, 36]. CATs could decompose hydrogen peroxide (H_2_O_2_) into oxygen (O_2_) and water (H_2_O), protecting normal cells from excessive ROS-mediated oxidative damage [37] (Fig. 2). Nanozymes that mimic CAT activity have been recognized as therapeutic sensitizers in cancer treatments, particularly immunotherapy [38]. Producing O_2_ catalyzed by these nanozymes at tumor sites is essential, as it potentially overcomes hypoxia during tumor treatment [39]. For instance, within melanoma sites, CaO_2_/DOX@SiO_2_/DOX-MnO_2_ nanodots with CAT-mimicking activity catalyze the hydrolysis of oxygen-storing CaO_2_ and H_2_O_2_, significantly improving oxygenation to alleviate tumor hypoxia and improve chemo-immunotherapy sensitivity [40]. Furthermore, similarly to their natural counterparts, the enzymatic activity of CAT-like nanozymes can be affected by various environmental elements, such as temperature (T), pondus hydrogenii (pH), and compounds in TME [41].

To date, nanomaterials incorporating metals such as zirconium (Zr), gold (Au), platinum (Pt), and iron (Fe) have been widely used to improve ROS generation such as hydroxyl radicals (·OH), ^1^O_2_, and superoxide anion (·O_2_^-^)[21, 42]. Various strategies have been developed to activate the decomposition of 2,2′-azobis[2-(2-imidazolin-2-yl)propane] dihydrochloride (AIPH), producing free alkyl radicals that synergize with the ROS generated to elevate oxidative stress levels [43]. The ZrO_2_-x@Pt/AIPH CAT-like nanoagent (ZPA) was synthesized by loading AIPH onto the surface of a Pt-ZrO_2_ nano-sonosensitizer. ZPA exhibits several advantages, including tumor-targeting properties, extended blood circulation, and stable sensitization performance. Under sonodynamic/thermodynamic conditions, ZPA significantly enhances intracellular oxidative stress, characterized by improved ICD and immune checkpoint blockade (ICB) sensitivity in mice bearing 4T1 tumor [44]. Similar findings by Wu et al. [45] demonstrated that photothermal-augmented ROS generation using CAT/POD-like Bi_2_Te_3_-Au/Pd nanosheets induces ICD and enhances therapeutic efficacy. Additionally, TiO_2_ nanospheres (HABT-C), possessing CAT/OXD activities derived from low-toxicity natural resources [46], effectively promote effector T cell infiltration into TME [47]. Furthermore, the mitigation of ROS scavenging by an Au/CuNDs-R848 nanovaccine enhances the immunogenicity of breast cancer cells, characterized by dendritic cell (DC) maturation, improved infiltration of T lymphocytes, and suppression of myeloid-derived suppressor cells (MDSCs). The CAT-mimicking activity of Au/CuNDs-R848 has been confirmed through its ability to produce ·OH in the presence of H_2_O_2_ [48]. A new CAT-like Fe-PDAP/Ce6 nanozyme, synthesized by loading the sonosensitizer agent Ce6 onto a cancer cell membrane (CCM)-coated Fe-PDAP, exhibits exceptional hypoxia relief capabilities and effectively generates ROS for sonodynamic immunotherapy against breast cancer. During sonodynamic therapy (SDT), the cancer cells produce ROS and release tumor-associated antigens (TAAs), which in turn promote DC maturation and T cell activation [49]. These studies support the crucial role of designing nanozymes with high catalytic efficiency for ROS production to enhance the sensitivity of immunotherapy in cancer treatment [50].

However, stress-induced ROS accumulation also stimulates tumorigenesis and therapeutic resistance by influencing various signaling pathways. Studies have highlighted the regulatory roles of cellular ROS in creating an immunosuppressive microenvironment, which enhances infiltration of myeloid components, such as MDSCs and tumor-associated macrophages (TAMs). A ROS scavenging nanozyme, Zr-CeO, was recently identified, exhibiting both CAT- and SOD-like activities. Administration of Zr-CeO inhibits MDSC recruitment and M2-type TAM polarization by dephosphorylating extracellular regulated protein kinases (ERK) and signal transducer and activator of transcription 3 (STAT3), leading to irreversible tumor cell death and increased sensitivity to PD-1 inhibition in renal subcutaneous tumors [51]. Ma et al. [52] synthesized a nanozyme, RhRe, and demonstrated its remarkable ability to scavenge ROS and generate O_2_, confirming its effective CAT enzyme activity. In particular, RhRe treatment, characterized by high biocompatibility and negligible toxicity, significantly suppresses tumor recurrence and inflammatory response in 4T1 tumor models. These unexpected findings may be attributed to restricted mitochondrial fission during stress conditions, contributing to nanozyme-induced ROS down-regulation and cell death in some cancer cells [53].

In addition, some CAT-like nanozymes can alleviate hypoxia, notably by enhancing the activity of glucose oxidase (GOx). Recently, engineered cascade nanocatalysts, such as AuPtAg-Gox [54] and gCM@MnAu [55], have been designed to modulate the host immune response. By amplifying GOx activity, these cascade nanocatalysts significantly reverse the immunogenically 'cold' tumor microenvironment, consequently increasing immunotherapy sensitivity in breast cancer. Similarly, a recent report by Wang et al. [56] introduced a hybrid nanocomposite material, CuCo(O)@PCN, which exhibits the CAT-like and GOx-like activities. Covalently linked to GOx, CuCo(O)@PCN enhances the enzymatic activity. By reacting with H_2_O_2_ to rapidly generate abundant O_2_, treatment with CuCo(O)@PCN has expectedly triggered robust anti-tumor immunotherapy sensitivity, characterized by increased intratumoral infiltration of pro-inflammatory cytokines and cytotoxic T cells.

### 2.2. Peroxidase-like nanozymes

The POD-mimicking nanomaterials have been investigated for their possible functions in anti-cancer immune response, presenting promising avenues for innovative therapeutic strategies. Integrating biomaterials with POD-like activity is expected to enhance therapeutic outcomes in cancer patients [42]. Mimicking POD activity, the nano-immunoremodelers Cu-DBCO/CL [57] and BiF_3_@BiOI@Pt-PVP [58] catalyze H_2_O_2_ into non-toxic products like water (Fig. 2). This surge in ROS induces mitochondrial dysfunction and insufficient ATP supply, and simultaneously triggers ferroptosis, cuproptosis, and ICD. The Cu_2_-xSe nanozyme (CSN) with GPx-like activities, functioning as vaccination in situ, dramatically drives free radical generation and ICD, reducing tumor metastasis and recurrence in surgery-cured mice. Immune analysis reveals elevated infiltration of effector T cells, M1 macrophages and mature DCs after CSN treatment [59]. The TME-responsive multienzyme nanosystem UCNPs@Cu-Cys-GOx (UCCG), integrating POD- and glutathione oxidase (GSHOx)-mimicking enzymes, boosts ROS concentration at tumor sites and reverses the immunosuppressive environment. Combining UCCG-induced chemodynamic therapy (CDT) with PD-L1 antibodies significantly prevents tumor growth and metastasis [60]. Additionally, Cu^2+^-doped POD-like immuno-nanozymes, CpG/Cu-LDHs [61], and Cu-NS@UK@POx [62], induce ROS storms and enhance anti-tumor efficacy by synergizing immune remodeling with ICD.

Recent advances also reveal Fe-based nanoparticles with intrinsic POD-like functions [63, 64] that specifically evoke anti-cancer immunity [65]. Fe-doped MOF (FessMOF) nanoparticles display excellent POD-like activity by breaking down H_2_O_2_ into ROS [66]. Light-triggered nanozyme Fe-TCPP-MOF with POD activity catalyzes cellular H_2_O_2_ decomposition, producing O_2_ to prompt immunogenic tumor cell death [67]. Similarly, a tirapazamine-loaded FessMOF nanoplatform, PP3244@Fe-ZT, exhibits specific cancer cell-targeting properties and immunomodulatory capacity. This POD-mimicking nanoplatform effectively catalyzes intracellular H_2_O_2_ to generate excessive free radicals, leading to cell ferroptosis and anti-cancer immunity [68]. These studies highlight widely applicable strategies for designing POD-like nanozymes to effectively modulate the immune response against cancer.

### 2.3. Oxidase-like nanozymes

Natural oxidase enzymes (OXD), such as GOx, lactate oxidase (LOD), and nicotinamide-adenine dinucleotide phosphate (NADPH) oxidase, drive redox reactions using molecular oxygen as electron acceptors to generate H_2_O or H_2_O_2_ (Fig. 2). Mimicking OXD activity, several bionanomaterials have been developed to display catalytic activity for substrate oxidation. For instance, the acid-sensitive LOD/CAT co-loaded nanozyme BMCL continuously releases H_2_O_2_, generating O_2_ to relieve hypoxia and depletes lactate in TME. Administration of BMCL promotes cell apoptosis and activates anti-tumor immunity in CT26 colon cancer-bearing mice [69]. Another innovative development is the Fe_3_O_4_-based hollow nanocarrier Syr/LOD@HFN, which has been designed to achieve the combined effects of CDT, immunotherapy, and starvation therapy. The Syr/LOD@HFN nanozyme sustainably produces H_2_O_2_, consequently driving the oxidative stress response. This process remodels the immune microenvironment by restoring cytotoxic NK and T cells, and increasing anti-cancer M1 macrophages in tumor tissues [70]. Additionally, a hollow nanomaterial HCS-FeCu with exceptional OXD/CAT/POD-mimicking catalytic activity has been engineered to enhance ROS production efficiency, induce photothermal-activated pyroptosis, and sensitize breast cancer to immunotherapy [71]. Zhang et al. [72] reported a hybrid nanozyme, Fe_3_O_4_@ZIF-8/GOx@MnO_2_. In this nanosystem, the acidic microenvironment promotes the breakdown of ZIF-8 layer, resulting in the release of GOx, MnO_2_, and Fe_3_O_4_. This process reshapes the immunosuppressive microenvironment by upregulating cellular levels of H_2_O_2_ and O_2_. The design of Fe-based nanoplatforms could inspire the application of OXD-like nanozymes in synergizing multiple anti-cancer therapeutic strategies, such as CDT and immunotherapy [72, 73]. Therefore, OXD nanozymes, characterized by high catalytic sustainability and stability, might offer attractive alternatives to natural enzymes in regulating immune homeostasis.

### 2.4. Superoxide dismutase-like nanozymes

SOD-mimicking nanozymes are critical in cancer immunity and immunotherapy, replicating the function of natural SOD enzyme, which converts ·O_2_^-^ into O_2_ and H_2_O_2_ (Fig. 2). These nanozymes help mitigate oxidative stress by reducing ROS levels in TME, thus restoring immune homeostasis, alleviating tumor-induced immunosuppression, and enhancing therapeutic efficacy [74]. Unlike natural enzymes, SOD-like nanozymes often exhibit multiple anti-oxidant activities and can more efficiently remove ROS during oxidative stress [75]. In particular, the metal single-atom nanozyme Fe-Cu-N6 shows exceptional selectivity toward SOD-like activity, seven times more effective than natural POD [76]. Recently, several novel cascade nanoimmunomodulators, such as CMZM [77] and IMZF [78], which possess multi-enzymatic activities including SOD, CAT, POD, and OXD, have been proposed to enhance cancer immunotherapy. CMZM demonstrates remarkable multi-enzyme-like activities that effectively counter the inhibitory tumor immune microenvironment. Through promoting DC maturation and effector T-cell infiltration, CMZM significantly activates anti-cancer immune response, providing a robust method to improve the outcomes of immunotherapy [77]. Similarly, IMZF administration promotes M2 to M1 macrophage polarization, reversing immune suppression [78]. Thus, integrating SOD-like nanozymes into cancer treatment promises to advance the effectiveness of immunotherapy by combining the benefits of ROS control with immune modulation. These innovative approaches represent the exciting frontier in developing next-generation cancer therapies.

## 3. Nanozyme-based technologies for cancer diagnosis

Nanozyme-based strategies have shown great promise for cancer diagnosis due to their unique characteristics and diverse applications. Nanozyme-based sensors can detect a variety of cancer biomarkers, such as nucleic acids, proteins, and small molecules. For instance, a platinum nanozyme Pt@CP has been successfully developed to provide a highly sensitive and high-throughput strategy for profiling proteins in urinary extracellular vesicles (uEV). By identifying three uEV biomarkers (mucin-1 (MUC-1), coiled-coil domain containing 25 (CCDC25), and solute carrier family 2 member 1 (GLUT1)), Pt@CP-based immunoassays are able to detect bladder cancer with high clinical specificity and sensitivity [79]. In addition, combining nanozymes with other advanced technologies, such as imaging devices, could create more powerful diagnostic tools. Mei et al.[80] developed a multifunctional metal-based nanozyme, CeAIP, for computed tomography/photothermal imaging-guided diagnosis of human cancers. CeAIP demonstrates exceptional biocompatibility and high accumulation at tumor sites, providing dual-mode imaging guidance for diagnosis of colon cancer.

## 4. Design of immune-associated nanozymes for improved prognosis of cancers

Cancers, when detected early, may be successfully and potentially cured. However, clinical management of patients with advanced cancer remains highly challenging due to the risk of recurrence and metastasis. Recently, advancements in nanotechnology, including nanozymes, have emerged as a potential solution for conducting more accurate and timely evaluations of cancer recurrence. This approach display the potential to enhance patient outcomes and increase efficiency of cancer treatment [81]. The multi-enzymatically-active nanocatalyst copper -quinone-GOx (CQG) has been designed to trigger ICD effect that combines pyroptosis and cuproptosis. This dual mechanism effectively eradicates dormant tumor cells and reduces the risk of cancer recurrence. By mimicking GPx and CAT activities, CQG generates a substantial amount of •OH radicals and oxygen. This process significantly disrupts the antioxidant defense mechanisms in cancer cells, particularly through the downregulation of NAD(P)H quinone dehydrogenase 1 (NQO1) signaling pathway. Meanwhile, CQG also exhibits SOD- and POD-like activities, rapidly enhancing cellular ROS levels. With its multi-enzyme-like catalytic activities, CQG is anticipated to combat tumor dormancy, prevent recurrence, and improve clinical outcomes [82]. Possessing immunological memory functions, the Mn-NP nanoenzyme-GOx hybrid (GOx-Mn/HA) significantly inhibits cancer recurrence and metastasis, leading to an extended survival time [83]. Furthermore, nanoenzyme-like hemostatic matrix system Surgiflo@PCN can effectively prevent postoperative bleeding in the tumor cavity, improving the prognosis [84]. These innovative engineering of nanomaterials to reprogram immune microenvironment and trigger robust immune response provides a groundbreaking strategy for addressing tumor dormancy and preventing relapse, thereby enhancing the efficacy of anti-tumor therapies.

Anti-cancer methods often fall short of controlling the disease effectively, as cancer cells can develop resistance to therapeutic agents, leading to adverse effects such as myelosuppression [85]. Therefore, developing innovative therapeutic modalities remains a critical focus of research to improve cancer treatment. During H_2_O_2_-induced stress, the CAT/POD-like nanoplatforms, CM@Mn [86] and FeCu-DA [87], were constructed to generate cellular ROS, subsequently enhancing immunotherapy sensitivity. Enzyme-like nanomaterials significantly improve tumor infiltration of effector immune cells, such as chimeric antigen receptor-natural killer (CAR-NK) cells [88], effector T cells [89], and M1-like TAMs [90, 91], consequently strengthening cancer immunotherapy by alleviating immunosuppressive microenvironment. In addition, IR780/CHCP nanozyme exhibits remarkable tumor growth inhibition and improves sensitivity to photothermal/photodynamic synergistic therapy [92]. Moreover, the photosensitive nanozymes could also improve the infiltration of cytotoxic T cells into tumor tissues, and enhance cancer immunotherapy [93]. P@Fe SAZ, the Fe-based single atom nanozyme, could remodel the immunosuppressive microenvironment, consequently boosting anti-tumor immunity and sensitivity of radiofrequency dynamic therapy [94]. Another POD-mimic Fe-based nanozymes, HCFe [95] and Fe SAZs [96], actively target tumor sites and lead to chemosensitization. More importantly, nanozymes exhibit remarkable biocompatibility and biodegradability, making them as promising candidates as therapeutic sensitizers against cancers [97, 98]. These findings provide new strategies for fabricating nanozyme-based technologies to overcome therapeutic limitations and hold promising potential in treatment sensitization. However, the long-term effects *in vivo* must be further confirmed.

## 5. Perspectives of nanozyme-based strategies in cancer immunotherapy

### 5.1. Utilizing microbe-based methods for improving nanozymes

The microbiome exhibits a broad spectrum of essential functions for human health, including immunity regulation, supporting the potential use of engineered microbes for therapeutic purposes [99, 100]. Moreover, microbe-based delivery methods show significant promises for enhancing the efficacy of nanozymes in therapeutic applications. The inherent advantages of bacteria combined with the unique properties of nanozymes present an innovative and potentially transformative approach for treating various diseases, particularly cancer. Bacteria/nanozyme composites provide synergistic cytotoxic effects against human diseases [101]. Engineered bacteria can specifically target tumors, facilitating precise nanozyme delivery to the desired site, enhancing therapeutic efficacy, and minimizing off-target effects. For instance, MnO_2_ nanoenzyme encapsulated in bacterial-derived vesicles proficiently converts H_2_O_2_ to O_2_, thereby alleviating oxidative stress and augmenting the anti-cancer efficacy of metalloimmunotherapy [102]. Au-Pt-based bimetallic vector Au-Pt@VNP20009, a novel bacterial/nanozyme system, was developed for intravenous injection. This system delivers micron-sized bacterial vectors to tumor sites via *in vivo* hitchhiking of CD11b+ immune cells. Biochemical experiments demonstrated that APV exhibits distinguished tumor-targeted killing ability and radio-immunotherapy sensitization, reducing tumor hypoxia and immunosuppression [103]. The pyroptosis inducer, LPZ, was constructed by integrating lactobacillus rhamnosus GG (LGG) and POD-like MOFs. Owing to the interaction between LGG pilin and cancer-associated mucins, LPZ can effectively bind to CCM and disrupt the membrane integrity, ultimately inducing pyroptotic cell death in 4T1 cells by elevating intracellular ROS concentration. This self-adaptive LPZ also induces intense immune activation to restrict the growth and metastasis of 4T1 cells [104]. However, comprehensive preclinical and clinical studies are imperative to ensure the safety and efficacy of these approaches for human application. Continued research and development are essential to fully realize the potential of bacterial-based nanozyme delivery systems.

### 5.2. Bioimage-based nanozymes in anti-cancer immunity

Incorporating imaging probes into nanozymes expands their clinical applications beyond tumor immunotherapy [105]. Bioimage-based nanoparticles offer dual benefits: facilitating tumor imaging modalities and stimulating robust anti-cancer immunity [106, 107]. Manganese dioxide (MnO_2_) is a promising candidate probe for magnetic resonance imaging (MRI), showing potential in cancer bioimaging and immune response evaluation [108]. Huang et al. [109] constructed a NK cell membrane-camouflaged Au@Pd@MnO_2_ nanostructure for MRI-guided multi-modal treatment, integrating photothermal therapy (PTT), CDT, and chemo-immunotherapy. This structure provides TME-responsive MR and NIR thermal imaging and exhibits CAT/POD-like activities. Camouflage with NK cell membrane enhances tumor targeting, immune evasion capabilities, and reliable tumor uptake. Moreover, the integration of polyethylene glycol (PEG) and tumor-targeting arginine-glycine-aspartic peptides (RGD) into nanozymes further enhances their targeting capacity, serving as attractive nanoprobes for monitoring bioimaging-guided anti-tumor immunity [110]. The multi-mode biosensing platform, Ab2@Au@Co_3_O_4_/CoFe_2_O_4_, exhibits excellent POD-like catalytic performance and electrochemiluminescence characteristics. Functioning as stable and reliable immunosensors, they provide a convenient and visible approach to detecting TAAs [111]. Similarly, super-paramagnetic iron oxide/heptamethine cyanine dye IR780-loaded nanocrystals offer outstanding MRI imaging and NIR fluorescence, providing a dual-modality imaging tool to visually detect the anti-tumor immune response [112]. Thus, with advances in imaging technology, nanozymes incorporating imaging probes are poised to enable visually guided anti-cancer immune responses.

### 5.3. Extracellular vesicle-based nanozymes

Extracellular vesicles (EVs) are gaining traction in cancer research for their distinguished diagnostic and therapeutic potential. These tiny, cell-secreted nanoparticles encapsulate diverse biomolecules, including nucleic acids, proteins, and lipids, which could all serve as cancer biomarkers [113-115]. The fusion of nanozymes with EVs has recently demonstrated considerable promise in boosting the accuracy and precision of cancer diagnostic methods. Recently, Huang and colleagues [116] designed a biomimetic nanozyme system (CF) by coating pyrite onto exosomes derived from tumors. The CF nanosystem significantly catalyzes redox imbalance and mitochondrial destruction, thereby overcoming the radiotherapy resistance in breast cancer cells. Meanwhile, uEVs-based immunoprobes could be utilized to discover the postoperative changes and residual tumors, facilitating prediction of cancer recurrence [79]. By coating AuNP-embedded nanozymes with tumor cell-derived exosome membrane, the multifunctional system CuPy-Au@EM achieves enhanced sensitivity, specificity, and rapid localization to the tumor sites. Utilization of CuPy-Au@EM together with low-dose radiotherapy triggers a highly effective anti-tumor response [117]. In summary, EV-based nanozymes offer a novel avenue for diagnosis and treatment of cancers. However, their application in anti-tumor immunity is still in its early stages. Ongoing research should focus on expanding their potential applications in immunotherapy regulation.

## 6. Clinical perspectives of nanozymes in cancer immunotherapy

The clinical application of nanozymes in cancer treatment is often limited by drawbacks such as relatively low catalytic activity, limited targeting capabilities, and systemic toxicity [118]. Furthermore, several nanozyme-based therapeutic strategies have failed clinical trials due to unsatisfactory effects, indicating that improvements in materials and technologies are required for effective clinical translation. By integrating steric restrictions and site-selective growth techniques, Ma’s group [119] created a distinctive pushpin-shaped nanozyme Au/CeO_2_, which exhibits elevated targeting effects and catalytic activity against tumors. In cancer cells, Au/CeO_2_-mediated ROS generation drives mitochondrial dysfunction and proteasomal damage, leading to oxidative stress and activation of innate immunity. A new cold-activated artificial enzyme, Bi_2_Fe_4_O_9_, was recently synthesized to mimic the GSHOx catalytic activity. Serving as in-situ vaccine, Bi_2_Fe_4_O_9_ has the ability to induce robust anti-cancer immunity and drive cold-enzymatic cell death (apoptosis and ferroptosis) with minimal off-target toxicity [120]. A bio-adhesive injectable hydrogel has been designed to enable the localized release of single-atom nanozyme (SAN) directly within tumor tissues. This approach minimizes systemic exposure, reduces toxicity, and protects SAN from degradation, ensuring more effective catalytic immunotherapy [121]. When composing heat-sensitive material cores, the nanozyme exhibits near-infrared (NIR)-controlled photoactivatable immunotherapeutic effects against cancers [31, 122].

**Figure 3.**
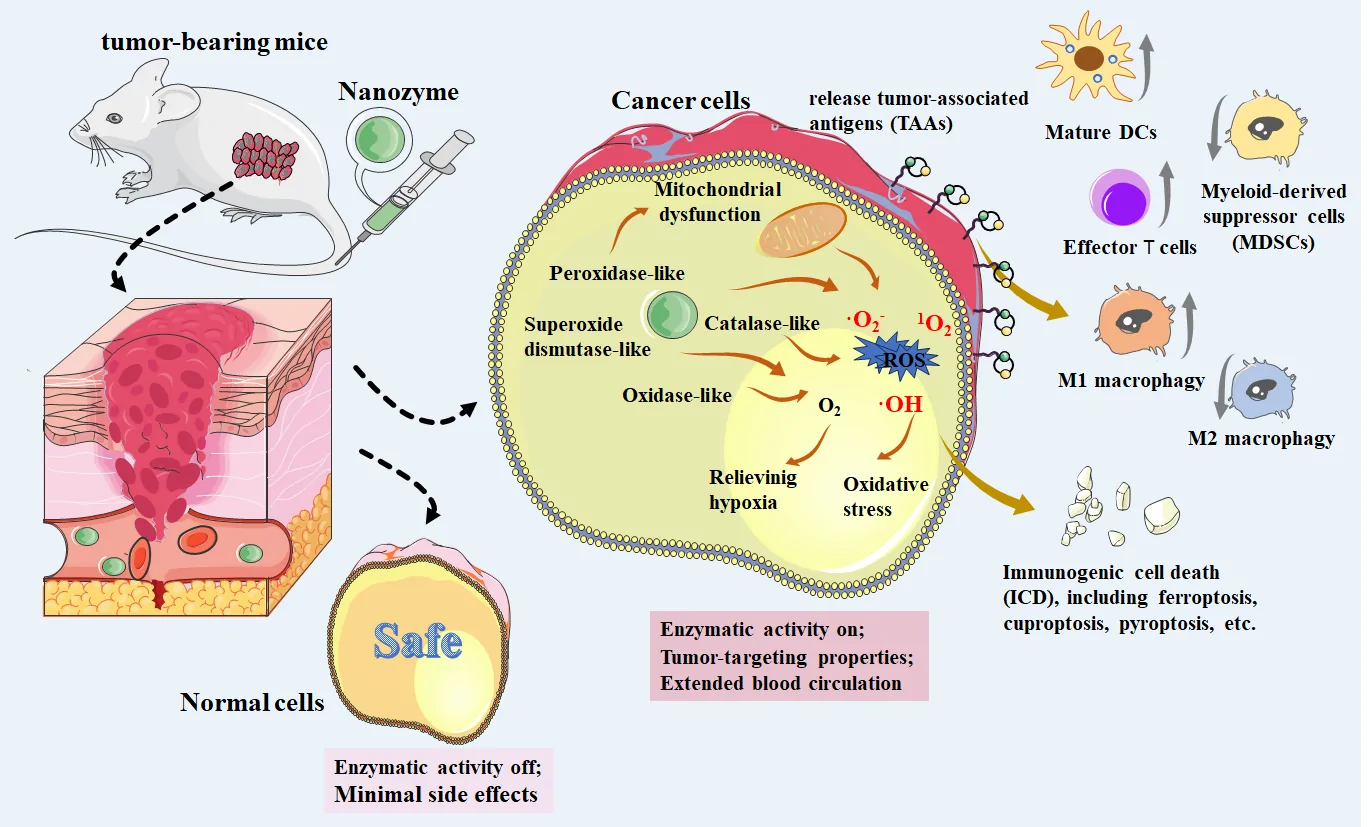
**The underlying mechanisms of catalytic nanosystems inducing anti-cancer immune response**. Nanozymes that mimic the activity of natural enzymes have been recognized as therapeutic sensitizers in anti-cancer immunity. Through regulating ROS homeostasis, oxidative stress and relieving hypoxia, tumor-targeting nanozymes enhance the anti-tumor immunity characterized by remodeling immune-cancer microenvironment and inducing ICD. In addition, nanozyme-driven immune responses represent an innovative therapeutic modality with minimal side effects.

Furthermore, optimizing the physicochemical properties, such as surface property regulations, affects biological effects of nanozymes, including cellular uptake and cytotoxicity. For example, compared to mesoporous spherical-like nanostructures, flower-like nanozymes exhibit superior enzyme-like activity and higher absorption efficiency by cancer cells [123]. Hyaluronic acid (HA) has been used to enhance dispersion stability of HABT-C nanozymes under physiological conditions [47]. Thus, optimizing these properties is crucial to improving the clinical translation potential of nanozymes in anti-tumor strategies. Given their high biosafety and robust efficacy [124] (Fig. 3), these novel nanocatalysts integrated with stimuli-responsive components could provide promising solutions for customized immunotherapy options.

In prodrug activation, bioorthogonal catalysis using transition metal catalysts (TMCs) provides a novel strategy by employing chemical reactions beyond the capabilities of natural enzymes. Although utilization of TMCs is challenged by some disadvantages, such as limited solubility, poor biocompatibility, and low stability in biological environments [125], these issues can be satisfactorily addressed by incorporating TMCs into nanozymes. This approach enhances catalytic activity, solubilizes TMCs, and protects them from deactivation, thus enabling flexible and sustained therapeutic production for cancer eradication. Nanozyme systems exhibit attractive biodegradability, facilitating their elimination through urine and feces [126]. Studies by Zhang et al. [127] revealed that bioorthogonal nanozymes display improved biocompatibility and stability. These nanozymes can be taken up by macrophages through endocytosis, which in turn activates the agonists of toll-like receptor 7/8 (TLR7/8) within the cells. This mechanism helps to reduce non-specific drug leakage and effectively activates the innate immune system to fight against cancer. Additionally, camouflaged nanoparticles can bypass immune detection, extend circulation time, and improve biocompatibility [128, 129]. For example, a biomimetic nanozyme coated with cell membrane showed high stability and cellular distribution, stimulating effective anti-tumor immunity by activating tumor-specific response and suppressing immune resistance [130]. Moreover, cell membrane-coated redox nanozyme CMO-R@4T1 functions as controlled therapeutic nanosystem to obtain long-term anti-cancer immunological memory [131]. The CD47-expressed cell membrane generates "don't eat me" signals, thereby preventing macrophage-mediated phagocytosis. This action subsequently prolongs blood stability and enhances bioavailability. Nanoparticles coated with CD47-positive CCM show an extended circulatory stability and induce targeted cytotoxicity against breast cancer [132]. The GOx-mimicking biomimetic nanozyme, AuNR@CeO_2_/AuNP-JQ1/RhB@M, enables targeted tumor bioimaging via fluorescence and induce cell pyroptosis in 4T1 tumor models [133].

## 7. The limitations of nanozyme-related technologies in cancer immunity

Although nanozyme-based cancer immunotherapies hold great promise, they still face several limitations. The biosafety of nanozymes is a crucial issue. The characteristics, such as size, morphology, surface atomic number, and surface charge, can significantly impact their bioactivity, biocompatibility, and toxicity. Achieving the satisfactory biosafety and specificity can be realized through rationally designing and optimizing the composition and structure of nanozymes [134, 135]. The second challenge is to elucidate the exact functional mechanisms of nanozymes, especially under the variable conditions of microenvironment. When assessing the potential for cancer management, it is crucial to consider the key factors that influence the catalytic behaviors and kinetics of nanozymes, such as the Michaelis-Menten model [136, 137]. In addition, how to maintain activity of nanozymes in complex microenvironment is another significant challenge [138]. Qin’s group [139] revealed that the acidity and high levels of glutathione (GSH) in microenvironment definitely weaken the anti-tumor activity of nanozymes. Even so, nanozymes display great capacity to make contributions to cancer management and serve as effective therapeutic agents for both standalone and combination cancer immunotherapy.

## 8. Conclusion

This review has outlined recent advances in nanozyme-based cancer immunotherapy strategies, emphasizing the potential opportunities and challenges faced in clinical applications. Anti-cancer therapies often grapple with issues such as low efficacy, therapeutic resistance, and unexpected side effects. Specific catalytic nanomaterials, characterized by tunable catalytic activities, multifunctionality, and enhanced tissue penetration, present promising alternatives and adjuncts for cancer management. Nanozymes have considerable potential to enhance anti-cancer immune response by remodeling immune microenvironment and effectively targeting tumor cells. Developments in nanotechnology have facilitated the use of nanozymes as carriers for catalytic drug delivery, enabling innovative synergistic approaches that augment traditional cancer treatments. Continued advancements in nanozyme technology could markedly improve existing methodologies and potentially revolutionize the clinical landscape for anti-cancer immunity.
